# Association Between Peripheral Blood Monocyte Count and Mucosal Healing in Japanese Patients With Ulcerative Colitis

**DOI:** 10.14309/ctg.0000000000000429

**Published:** 2021-11-18

**Authors:** Shinya Furukawa, Yoshio Ikeda, Sen Yagi, Teruki Miyake, Kana Shiraishi, Kazuhiro Tange, Yu Hashimoto, Kenichirou Mori, Tomoyuki Ninomiya, Seiyuu Suzuki, Naozumi Shibata, Hidehiro Murakami, Katsuhisa Ohashi, Aki Hasebe, Hideomi Tomida, Yasunori Yamamoto, Eiji Takeshita, Yoichi Hiasa

**Affiliations:** 1 Health Services Center, Ehime University, Ehime, Japan;; 2 Endoscopy Center, Ehime University Hospital, Ehime, Japan;; 3 Department of Internal Medicine, Saiseikai Matsuyama Hospital, Matsuyama, Ehime, Japan;; 4 Department of Gastroenterology and Metabology, Ehime University Graduate School of Medicine, Ehime, Japan;; 5 Department of Inflammatory Bowel Diseases and Therapeutics, Ehime University Graduate School of Medicine, Ehime, Japan;; 6 Department of Gastroenterology, Ehime Prefectural Central Hospital, Matsuyama, Ehime, Japan;; 7 Department of Gastroenterology, Sumitomo Besshi Hospital, Niihama, Japan;; 8 Department of Gastroenterology, Ehime Prefectural Niihama Hospital, Niihama, Ehime, Japan;; 9 Ohashi Clinic Participate in Gastro-Enterology and Ano-Proctology, Niihama, Ehime, Japan;; 10 Department of Gastroenterology, Shikoku Cancer Center, Matsuyama, Ehime, Japan.

## Abstract

**INTRODUCTION:**

Monocytes play an important role in innate immunity. Some epidemiological evidence indicates an association between peripheral blood monocytes and ulcerative colitis (UC). The association between peripheral blood monocytes and mucosal healing (MH), however, remains unclear. We evaluated this issue in patients with UC.

**METHODS:**

Study subjects consisted of 272 Japanese patients with UC. Monocyte counts were taken in the morning after overnight fasting. Monocyte count was divided into tertiles based on the distribution of values among all study subjects. Information on clinical remission was obtained from medical records. MH was assessed using the Mayo endoscopic subscore.

**RESULTS:**

The mean monocyte count was 360.1 ± 155.3/mm^3^. Rates of clinical remission, MH, and complete MH were 61.0%, 66.2%, and 27.9%, respectively. High monocyte count was significantly inversely associated with clinical remission, MH, and complete MH (adjusted odds ratio [OR] 0.45 [95% confidence interval [CI]: 0.23–0.89], OR 0.45 [95% CI: 0.23–0.89], and OR 0.48 [95% CI: 0.23–0.97], respectively). Patients were also classified according to C-reactive protein (CRP) levels; in the low CRP group (<0.1 mg/dL), high monocyte count was independently inversely associated with complete MH but not with clinical remission or MH (OR 0.33 [95% CI: 0.10–0.92], *P* for trend = 0.027). In the high CRP group, there was no association between monocyte count and clinical outcomes.

**DISCUSSION:**

Our findings suggest that peripheral blood monocyte count can be used as a serum supplemental marker for MH in UC patients with low CRP levels.

## INTRODUCTION

The clinical course of ulcerative colitis (UC) is characterized by repeated relapses and remissions (1). Mucosal healing (MH) is inversely associated with clinical relapse, rates of hospitalization and surgery, and incidence of colorectal cancer (2–7); accordingly, MH is the therapeutic goal for UC. As the number of patients with UC increases in Asian countries and remains high in Western countries (8), the unmet need for a rapid, repeatable, cost-effective, and clinically useful serum biomarker for MH, a necessary key to achieving long-term MH in patients with UC, grows more urgent worldwide (9).

Peripheral blood leukocyte subtypes including monocytes can be monitored at clinical visits; such data are routinely available. Dysregulation of mucosal innate immunity seems to be a key factor in the pathogenesis of UC (10), and monocytes play an important role in innate immunity. There are several sources of evidence regarding the association between peripheral blood monocyte count and disease activity in patients with UC (11–15). An association between peripheral blood monocyte count and MH in patients with UC, however, is yet to be confirmed.

In general, serological biomarkers for intestinal inflammation have lower sensitivity and less specificity in patients with UC compared with those in other patients (16). We have previously reported that, in patients with UC, the expected inverse association between serum globulin level and MH was found only in a low-C-reactive protein (CRP) group and not in a high-CRP group (17). Among patients with colorectal cancer, the combination of CRP and monocyte count is a useful marker for prognosis (18). Thus, we hypothesize that the combination of CRP and peripheral blood monocyte count might be a more useful prognostic marker for clinical outcomes in patients with UC as well. In this study, we explored the association between peripheral blood monocyte count and clinical outcomes including MH after CRP stratification in Japanese patients with UC.

## METHODS

### Study population

Study subjects in the baseline survey consisted of 387 Japanese patients with UC who visited the Department of Gastroenterology and Metabology at the Ehime University Graduate School of Medicine or several affiliated hospitals in Ehime Prefecture. All patients who were able to consent to the study and respond to the self-administered questionnaire were considered as candidates for inclusion. The final analysis sample in this study consisted of 272 patients because 115 patients were excluded because of incomplete data. All patients had been diagnosed with UC based on radiological, endoscopic, histological, and clinical criteria. This study was conducted in accordance with the Declaration of Helsinki, and the study protocol was approved by the institutional review board of the Ehime University Graduate School of Medicine (#1505011). Trained staff obtained written informed consent from all enrolled patients. This study cohort was registered during the period 2015–2019.

### Measurements

Information on CRP, peripheral blood monocyte count, endoscopic findings, disease extent, clinical remission, and the use of medication for UC was obtained from medical records. Other variables were reported in a self-administered paper-and-pencil questionnaire. Blood samples were taken in the morning after overnight fasting. A well-trained laboratory medical technologist measured each patient's monocyte count using a flow cytometer. Height was measured to the nearest millimeter using a stadiometer with the patient standing completely erect. Weight was measured in light clothing. Body mass index (BMI) was calculated as weight in kilograms divided by height in meters squared. Blood examination was performed when a colonoscopy was booked or when the colonoscopy examination was performed, and up to 2 months may have passed between the blood examination and the colonoscopy.

#### Assessment of clinical remission, endoscopic activity, and MH.

Clinical remission was defined as no rectal bleeding and stool frequency < 3 times per day.

MH was assessed using the Mayo endoscopic subscore (MES) (19). In this study, MH and complete MH were defined as categories 0 and 0–1, respectively. All endoscopic findings were interpreted by only 1 endoscopy specialist, who was blinded to CRP and peripheral blood monocyte count.

#### Statistical analysis.

Monocyte count was divided into tertiles according to the distribution of this value among all study subjects as follows: (i) low monocyte count, < 281/mm^3^ (reference category); (ii) moderate monocyte count, 281–391/mm^3^; and (iii) high monocyte count, ≥ 391/mm^3^. CRP values were classified as either low (<0.1 mg/dL) or high (≥0.1 mg/dL). Crude odds ratios (ORs) and their 95% confidence intervals (CIs) for clinical remission, MH, and complete MH in relation to peripheral blood monocyte count were estimated using logistic regression analysis. Multiple logistic regression analyses were used to adjust for potential confounding factors. Age, sex, BMI, disease extent (proctitis/nonproctitis), prednisolone use, and antitumor necrosis factor (TNF)-α monoclonal antibody preparation use were selected *a priori* as potential confounding factors. All statistical analyses were mainly performed using SAS software package, version 9.4 (SAS Institute, Cary, NC). A receiver operating characteristic (ROC) curve was generated, and the area under the curve was calculated to measure the utility of monocyte count in indicating MH. JMP 14.2 (SAS Institute) was used to analyze the ROC curve and to identify the cutoff value, specificity, and sensitivity of peripheral blood monocyte count. All probability values for statistical tests were 2-tailed, and *P* < 0.05 was considered statistically significant.

## RESULTS

### Cohort characteristics

Table 1 summarizes the characteristics of the 272 study patients. The mean age, mean BMI, and proportion of male subjects were 51.0 years, 22.85, and 57.7%, respectively. The proportions of subjects treated with 5-aminosalicylates, prednisolone, anti-TNF-α monoclonal antibody preparations, and azathioprine were 91.2%, 19.9%, 5.5%, and 16.5%, respectively. The mean monocyte count was 360.1 ± 155.3/mm^3^. Peripheral blood monocyte count was significantly higher in patients treated with prednisolone than in patients not treated with prednisolone (436.0 ± 197.4/mm^3^ vs 341.4 ± 137.2/mm^3^, *P* = 0.001) but did not differ between patients treated with and without any other medications.

**Table 1.** Clinical characteristics of 272 study participants

Variable	n (%)
Age, yr, mean ± SD	51.0 ± 16.2
Male (%)	157 (57.7)
BMI	22.85 ± 4.59
Disease extent (pancolitis/left-sided/procitis/others)	115/71/79/7
Medication	
5-aminosalicylates (%)	248 (91.2)
Prednisolone (%)	54 (19.9)
TNF-α monoclonal antibody (%)	15 (5.5)
Azathioprine (%)	45 (16.5)
Clinical remission, %	166 (61.0)
MES, mean ± SD	1.12 ± 0.89
Partial MH (MES ≤ 1) (%)	180 (66.2)
MH (MES < 1) (%)	76 (27.9)
Monocyte counts,/mm^3^, mean ± SD	360.1 ± 155.3
CRP, mg/dL, median ± IQR	0.099 ± 0.21

BMI, body mass index; CRP, C-reactive protein; IQR, interquartile range; MES, Mayo endoscopic subscore; MH, mucosal healing; TNF, tumor necrosis factor.

### Association between peripheral blood monocyte count and clinical outcomes

Table 2 summarizes the association between peripheral blood monocyte count and clinical remission, MH, and complete MH. High monocyte count was inversely associated with clinical remission, MH, and complete MH (crude OR 0.36 [95% CI: 0.19–0.66], crude OR 0.37 [95% CI: 0.19–0.70], and crude OR 0.39 [95% CI: 0.20–0.75], respectively). Even after adjustment for confounding factors, high monocyte count was independently inversely associated with clinical remission, MH, and complete MH (clinical remission: adjusted OR 0.45 [95% CI: 0.23–0.89], *P* for trend = 0.036, MH: adjusted OR 0.45 [95% CI: 0.23–0.89], *P* for trend = 0.023, and complete MH: adjusted OR 0.48 [95% CI: 0.23–0.97], *P* for trend = 0.021).

**Table 2.** Crude and adjusted ORs and 95% CIs for the associations between monocyte counts and clinical outcomes

Variable	Prevalence (%)	Crude OR (95% CI)	Adjusted OR (95% CI)
Clinical remission			
Low (monocyte count <281/mm^3^)	65/90 (72.2)	1.00	1.00
Moderate (281 ≤ monocyte count <391/mm^3^)	57/91 (62.6)	0.65 (0.34–1.20)	0.70 (0.36–1.35)
High (391/mm^3^ ≤ monocyte count)	44/91 (48.4)	0.36 (0.19–0.66)	0.45 (0.23–0.89)
*P* for trend			0.036
Partial MH (MES ≤1)			
Low (monocyte count <281/mm^3^)	69/90 (76.7)	1.00	1.00
Moderate (281 ≤ monocyte count <391/mm^3^)	61/91 (67.0)	0.62 (0.32–1.19)	0.69 (0.35–1.36)
High (391/mm^3^ ≤ monocyte count)	50/91 (55.0)	0.37 (0.19–0.70)	0.45 (0.23–0.89)
*P* for trend			0.023
MH (MES < 1)			
Low (monocyte count <281/mm^3^)	35/90 (38.9)	1.00	1.00
Moderate (281 ≤ monocyte count <391/mm^3^)	23/91 (25.3)	0.53 (0.28–0.998)	0.57 (0.29–1.10)
High (391/mm^3^ ≤ monocyte count)	18/91 (19.8)	0.39 (0.20–0.75)	0.48 (0.23–0.97)
*P* for trend			0.021

ORs were adjusted for age, sex, body mass index, use of prednisolone, use of tumor necrosis factor-α monoclonal antibody, and disease extent.

CI, confidence interval; MES, Mayo endoscopic subscore; MH, mucosal healing; OR, odds ratio.

### Association between Peripheral blood monocyte count and MH after CRP stratification

**Figure 1.**
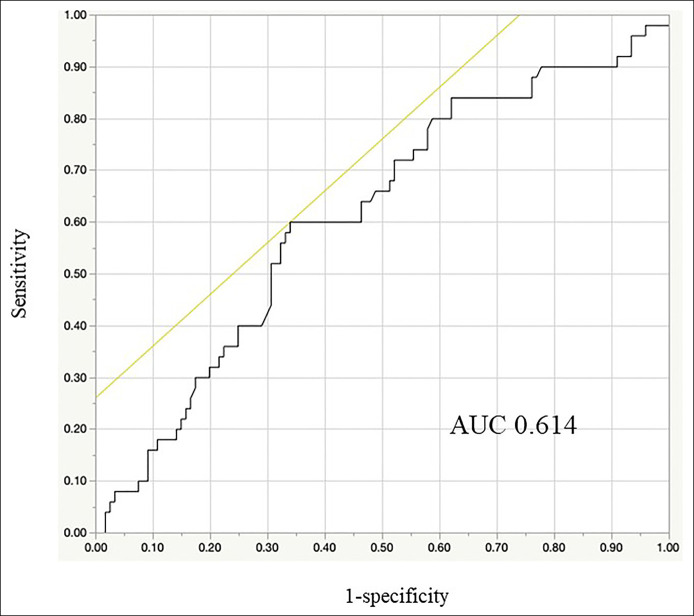
Receiver operating characteristic (ROC) curve showing the usefulness of peripheral blood monocyte count as a marker for complete mucosal healing (MH) in the low C-reactive protein group. The ROC curve for peripheral blood monocyte count as a marker for identifying complete MH had an area under the curve of 0.614. When the cutoff value of peripheral blood monocyte count was set to 285/mm^3^, the sensitivity and specificity of this marker were 60.0% and 66.1%, respectively.

Table 3 summarizes the crude and adjusted ORs and 95% CIs for clinical outcomes in relation to peripheral blood monocyte count after CRP stratification. In the low CRP group (CRP < 0.1 mg/dL), an inverse association between monocyte count and clinical outcome was found in the crude analysis. After adjustment, however, high monocyte count was independently inversely associated with complete MH (adjusted OR 0.33 [95% CI: 0.13–0.92], *P* for trend = 0.027), but not with clinical remission or MH. In the high CRP group (CRP ≥ 0.1 mg/dL), there was no association between peripheral blood monocyte count and clinical outcomes. In the low CRP group, the ROC curve of the peripheral blood monocyte count for identifying complete MH had an area under the curve of 0.614 (Figure 1).

**Table 3.** Crude and adjusted ORs and 95% CIs for the associations between monocyte count and clinical outcomes according to patient CRP level

Variable	Prevalence (%)	Crude OR (95% CI)	Adjusted OR (95% CI)
CRP < 0.1 mg/dL (n = 147)			
Clinical remission			
Low (monocyte count < 281/mm^3^)	44/61 (72.1)	1.00	1.00
Moderate (281 ≤ monocyte count < 391/mm^3^)	33/50 (66.0)	0.75 (0.33–1.69)	0.82 (0.34–1.97)
High (391/mm^3^ ≤ monocyte count)	18/36 (50.0)	0.39 (0.16–0.91)	0.53 (0.20–1.40)
*P* for trend			0.21
Partial MH (MES ≤ 1)			
Low (monocyte count <281/mm^3^)	50/61 (82.0)	1.00	1.00
Moderate (281 ≤ monocyte count < 391/mm^3^)	34/50 (68.0)	0.47 (0.19–1.12)	0.48 (0.19–1.19)
High (391/mm^3^ ≤ monocyte count)	23/36 (63.9)	0.39 (0.15–0.996)	0.52 (0.19–1.43)
*P* for trend			0.17
MH (MES < 1)			
Low (monocyte count <281/mm^3^)	26/61 (42.6)	1.00	1.00
Moderate (281 ≤ monocyte count < 391/mm^3^)	13/50 (26.0)	0.47 (0.21–1.05)	0.50 (0.21–1.15)
High (391/mm^3^ ≤ monocyte count)	6/36 (16.7)	0.27 (0.09–0.71)	0.33 (0.10–0.92)
*P* for trend			0.027
CRP ≥ 0.1 mg/dL (n = 125)			
Clinical remission			
Low (monocyte count <281/mm^3^)	21/29 (72.4)	1.00	1.00
Moderate (281 ≤ monocyte count < 391/mm^3^)	24/41 (58.5)	0.54 (0.19–1.47)	0.65 (0.21–1.94)
High (391/mm^3^ ≤ monocyte count)	26/55 (47.2)	0.34 (0.12–0.88)	0.44 (0.15–1.24)
*P* for trend			0.12
Partial MH (MES ≤ 1)			
Low (monocyte count <281/mm^3^)	19/29 (65.5)	1.00	1.00
Moderate (281 ≤ monocyte count < 391/mm^3^)	27/41 (65.9)	1.02 (0.37–2.76)	1.18 (0.39–3.62)
High (391/mm^3^ ≤ monocyte count)	27/55 (49.1)	0.51 (0.20–1.27)	0.62 (0.21–1.73)
*P* for trend			0.26
MH (MES < 1)			
Low (monocyte count < 281/mm^3^)	9/29 (31.0)	1.00	1.00
Moderate (281 ≤ monocyte count < 391/mm^3^)	10/41 (24.4)	0.72 (0.25–2.10)	0.88 (0.28–2.77)
High (391/mm^3^ ≤ monocyte count)	12/55 (21.8)	0.62 (0.23–1.74)	0.88 (0.29–2.73)
*P* for trend			0.83

ORs were adjusted for age, sex, body mass index, use of prednisolone, use of tumor necrosis factor-α monoclonal antibody, and disease extent.

CI, confidence interval; CRP, C-reactive protein; MES, Mayo endoscopic subscore; MH, mucosal healing; OR, odds ratio.

## DISCUSSION

This study demonstrates that peripheral blood monocyte count is independently inversely associated with clinical remission and MH among patients with UC. To the best of our knowledge, this is the first study to show an inverse association between peripheral blood monocyte count and MH in patients with UC. In the low CRP group, high monocyte count was independently inversely associated with MH, but not partial MH or clinical remission. In UC patients with low CRP, therefore, peripheral blood monocyte count might serve as a supplemental blood marker for MH.

Several previous studies have reported an association between peripheral blood monocyte count and UC. In a US case–control study of 260 subjects including 110 UC patients, as mentioned earlier, the monocyte count was higher in patients with active-stage UC than in those with remission-stage UC (11). In an Egyptian study of patients with UC, monocyte count was higher among patients in active stage (MES ≥2) than among those in remission stage (MES 0–1) (13). In a UK study of 139 subjects (83 patients with IBD, 37 healthy controls, and 17 disease controls), absolute peripheral blood monocyte count was higher in patients with UC than in healthy controls and was positively associated with disease severity of UC (12). In a US cohort study of 1,290 patients with IBD including 463 patients with UC (14), monocytosis was positively associated with CRP, anemia, disease severity, hospitalization, surgery, emergency department visits, and annual financial health-care costs. In addition, quality of life was worse in UC patients with concomitant monocytosis than in UC patients without it. The findings of this study are consistent with these previous studies, revealing various associations between peripheral blood monocyte count and clinical outcomes.

By contrast, a Chinese study of 217 patients with IBD (76 UC and 141 Crohn's disease) found that peripheral blood monocyte count did not significantly differ between active-stage and inactive-stage IBD, regardless of *Clostridioides difficile* infection, although a positive relationship between peripheral blood monocyte count and CRP was observed in patients with active UC (15). The discrepancy between this finding and our study results may be explained, partly, by differences in sample size, study design, assessment of disease activity, medication use, and patients' age.

Although we have shown a link between peripheral blood monocyte count and MH in patients with UC, the underlying mechanism linking peripheral blood monocyte count and MH remains unclear. Persistent activation of monocytes and dysregulation of innate immune responses are involved in the development of UC (20). Proinflammatory cytokines and chemokines can generate monocytes and recruit them to areas of inflammation. Peripheral blood monocyte and macrophage counts in the blood are linked to those in areas of inflammation (21). Excessive numbers of peripheral blood monocytes can impair healing in an ischemic heart disease model (21,22).

IL-17 also plays a pathological role in UC (23): IL-17A activates various cells including monocytes that release TNF-α, IL-1B, and chemokines (24). The association between proinflammatory cytokines (such as IL-6, IL-17, and TNF-α) and mucosal injury has been reported in patients with UC (25,26). Monocytes that have been activated by proinflammatory cytokines and chemokines might inhibit MH by elevating TNF-α, IL-1B, and chemokines. It remains to be seen whether abnormal peripheral blood monocyte counts are involved in the etiology of UC. In addition, further research is warranted to investigate possible associations between the extracellular matrix, proteases, and peripheral blood monocyte counts in patients with UC.

This study has some limitations. First, because a cross-sectional design was used, we cannot confirm a causal relationship between peripheral blood monocyte count and MH. Second, it is likely that most of the patients in this cohort had been receiving long-term treatment before the study. The long duration of treatment might have affected peripheral blood monocyte counts and endoscopic activity. Third, the possibility of other concurrent collagen diseases that might elevate CRP such as primary sclerosis cholangitis and rheumatoid arthritis was not excluded, and such diseases may have confounded the results. Fourth, CRP was measured using different kits in different patients, hospitals, and periods. In Japan, however, the measurement methods for common laboratory data, including CRP, are standardized, and all common laboratory data in this cohort are quality controlled using standard substances (27). Finally, the subjects of this study were not likely representative of Japanese patients with UC, although the median age, the male-to-female ratio, and the rate of prednisolone use in this study (48.0%, 57.7%, and 19.9%, respectively) were similar to those in a Japanese national study of 7,907 patients with UC (44.0%, 63.9%, and 15.5%, respectively) (28).

In conclusion, peripheral blood monocyte count may be significantly inversely associated with clinical remission, partial MH, and MH in Japanese patients with UC. In UC patients with low CRP, peripheral blood monocyte count might be significantly inversely associated with only MH. In UC patients with low CRP, therefore, peripheral blood monocyte count might serve as a supplemental blood marker for MH.

## CONFLICTS OF INTEREST

**Guarantor of the article:** Shinya Furukawa, MD, PhD.

**Specific author contributions:** Study conception and design: S.F., E.T., Y.I., and Y.H.: Material preparation and data collection: S.F., S.Y., K.S., K.T., Y.H., K.M., T.N., S.S., N.S., H.M., K.O., A.H., H.T., Y.Y., E.T., and Y.I.: Data analysis: S.F. Interpretation of data: S.F., T.M. and Y.I.: First draft writing: S.F. All authors revised and edited drafts and read and approved the final manuscript.

**Financial support:** None to report.

**Potential competing interests:** None to report.

**Study Highlights**

**WHAT IS KNOWN**

✓ The number of patients with ulcerative colitis (UC) is increasing.
✓ Mucosal healing (MH) is the therapeutic goal for UC.
✓ Monocytes play an important role in innate immunity.
✓ Some studies have shown an association between peripheral blood monocyte count and disease activity in patients with UC.
✓ The association between peripheral blood monocyte count and MH remains unclear.

**WHAT IS NEW HERE**

✓ Peripheral blood monocyte count is significantly inversely associated with clinical remission, MH, and complete MH in Japanese patients with UC.
✓ In patients with low C-reactive protein, peripheral blood monocyte count might be significantly inversely associated with only MH but not clinical remission.
✓ In patients with high C-reactive protein, no association between peripheral blood monocyte count and clinical outcomes was found.
